# Eco-friendly revenues for healthcare: assessing the relationship between green taxation, public health expenditures, and life expectancy in China

**DOI:** 10.3389/fpubh.2024.1358730

**Published:** 2024-05-22

**Authors:** Di Zhang

**Affiliations:** School of Finance and Taxation, Henan Finance University, Zhengzhou, Henan, China

**Keywords:** green taxation, public health expenditures, life expectancy, health economic, green economics

## Abstract

**Introduction:**

The synergy of green taxation, public health expenditures, and life expectancy emerges as a compelling narrative in the intricate symphony of environmental responsibility and public well-being. Therefore, this study examine the impact of green taxation on life expectancy and the moderating role of public health expenditure on the said nexus, particularly in the context of China, an emerging economy.

**Methods:**

Statistical data is collected from the National Bureau of Statistics of China to empirically examine the proposed relationships. The dataset contains provincial data across years.

**Results:**

Using fixed-effect and system GMM regression models alongwith control variables, the results found a positive and statistically significant influence of green taxation on life expectancy. Moreover, public health expenditures have a positive and statistically significant partial moderating impact on the direct relationship.

**Discussion:**

These findings suggest that the higher cost of pollution encourages individuals and businesses to shift to less environmentally harmful alternatives, subsequently improving public health. Moreover, government investment in the health sector increases the availability and accessibility of health facilities; thus, the positive impact of green taxation on public health gets more pronounced. The findings significantly contribute to the fields of environmental and health economics and provide a new avenue of research for the academic community and policymakers.

## 1 Introduction

The nexus between environmental policy and public health and its collective influence on human well-being has emerged as a prominent concern in today’s globalized and environmentally challenged world (1, 2). The use of environmental/green taxes as a prominent policy instrument has gained significant attention due to its potential to effectively tackle environmental degradation and encourage sustainable practices (3). Moreover, sufficient funding for public health expenditures could be an important factor in improving health outcomes and community well-being (4, 5). Therefore, the purpose of this study is to explore how life expectancy can be enhanced through green taxation and how public health expenditure can moderate the said life expectancy and green taxation nexus.

Green taxes are somehow a deliberate approach designed to motivate sustainable behavior and address the harmful effects of environmental consequences (6). This phenomenon indicates a growing global awareness of the detrimental effects of pollution and the reduction of natural resources (7). The purpose of these taxes is to create a framework for addressing environmental concerns that affect public health by internalizing external costs and providing incentives for environmentally responsible behavior (8, 9). Climate change, air and water pollution, and resource shortages all have long-lasting effects on the quality of life (10, 11); therefore, it’s important to understand how environmental taxes affect the welfare of societies.

Assessing life expectancy is a crucial component in evaluating the overall impact of environmental policy and public health spending (12, 13), making it a vital tool for defining the overall well-being and prosperity of a particular community (14, 15). Such a phenomenon can better describe the complex interaction of various factors that influence the overall welfare of individuals. These factors include the environment, individual lifestyle choices, aging, and the availability and accessibility of healthcare services (16, 17). Therefore, a study that investigates the relationship between green taxes and life expectancy can provide important insights regarding the effectiveness of environmental policies in improving public health outcomes.

Furthermore, it is vital to recognize the significance of public health expenditure, which links to the allocation of financial resources by the government toward healthcare services/infrastructure (18, 19). The moderating variable holds the capability to either increase or decrease the impact of green taxation on life expectancy. The negative health consequences resulting from environmental damage can be mitigated by providing adequate funding to the public health system (20–22). Consequently, this situation improves the efficacy of green taxes in attaining their planned objectives. It is crucial for efficient policymaking and resource optimization to understand how public health expenditures impact the nexus between green taxation and life expectancy (23, 24).

The main purpose of this research is to examine the impact of green taxation on life expectancy and the moderating role of public health expenditures, particularly within the context of China’s dynamic and rapidly growing economy. Thus, this study helps to address a critical gap in the literature. Through comprehensive analysis and the application of rigorous econometric testing, we aim to clarify the said nexus and offer new insights to policymakers for the design and delivery of an efficient public health policy. Moreover, the findings of this study encourage the development of policies to boost environmental sustainability and improve public health. Furthermore, these outcomes have relevance for individuals, societies, and organizations that are committed to advancing a more impartial and prosperous future for global societies.

This study focuses on the Chinese economy, where fast industrialization and urbanization have caused several environmental and health concerns; thus, examining green taxation, public health expenditures, and life expectancy is necessary (2). Green taxation encourages sustainable behaviors and reduces industrial pollution. Whereas, public health expenditures also need to be examined to assess the healthcare system for a growing population facing severe environmental risks (25). Policymakers can understand the complex relationships between environmental policies, the healthcare sector, and longevity by analyzing the link proposed in this study.

The rest of the paper is organized as follows: section 2 discusses the literature. Section 3 explains the background of the study. Section 4 entails the methodology to testify to the hypotheses of the study. Section 5 documents the empirical analysis and discussion. Lastly, Section 6 concludes the findings and provided policy implications, limitations, and future research directions.

## 2 Literature review

We are living in a time when the world is facing numerous health and environmental challenges. Therefore, fields like public health and environmental policy have gained significant attention. Several studies have been conducted to explore how life expectancy can be improved, and many determinants have also been investigated. Table 1 summarizes the recent literature on the topic.

**Table 1:** Literature review.

References	Findings
Wang and Tang (26)	The study examined the impact of air pollution and environmental levies on individual well-being and life expectancy. Using the CSS (China Social Survey) and addressing endogeneity problems, researchers discovered that air pollution can affect citizens’ well-being and that green taxes can not only increase residents’ well-being but also reduce the negative impact of air pollution on their well-being.
Arltová and Kot (27)	The authors researched the relationship between green taxation and environmental quality in OECD nations, and their findings showed that more taxation leads to improved quality, which ultimately enhances wellbeing and life expectancy.
Soku et al. (28)	The findings discovered that green taxes reduce carbon emissions and, as a result, improve lives and overall life expectancy.
Zhang et al. (5)	The research shows that environmental and green taxes could promote health outcomes and subsequently enhance life expectancy.
Wang et al. (29)	The authors discovered that green taxes significantly reduce environmentally destructive activities and, hence, improve life expectancy in BRICS countries.
Anwar et al. (30)	The study evaluated the impact of health spending on life expectancy in OECD countries. The findings discovered that health spending has a positive impact on life expectancy in the examined countries.
Awoyemi et al. (31)	This study investigated the effect of government health spending on life expectancy and death rates in Nigeria. According to the study, increased government health spending increases life expectancy and lowers the death rate.
Ahmad et al. (32)	The researchers discussed the impact of health spending on life expectancy in South Asian countries, as well as the function of health spending in moderating the relationship between industrialization, income disparity, and life expectancy. The findings demonstrated that health spending had a considerable impact on life expectancy in Southeast Asian countries.
Zhang et al. (5)	This study explored the nexus between environment-related governance, public health spending, and economic growth. The findings suggested that higher pollution can significantly reduce health and economic growth. Moreover, green or environmental taxes could improve health and life expectancy.
Lopreite et al. (16)	By conducting a bibliographic analysis, the authors explored that an increase in health spending is required to meet the aging requirement and access to healthcare services.
Lopreite and Zhu (15)	The authors examined the relationships between aging, life expectancy, health expenditure, and economic growth. The study compared the US and China, and the findings suggested that effective policies (economic, social, and health) are needed to improve the quality of life and achieve sustainable growth.
Behera and Dash (33)	The authors examined the impact of health expenditures on achieving healthcare goals in Southeast Asian countries. The results enlightened that health expenditure has a positive impact on the improvement in life expectancy.
Behera and Dash (34)	The paper studied the impact of macro-level fiscal policies on health financing in lower-middle-income countries. The findings suggested a positive impact of tax revenue on public health expenditures in the sample countries.
Lopreite and Mauro (22)	The study investigated the relationship between demographic changes and health expenditures, and the results found that expenditures on health have a more pronounced impact on aging as compared to life expectancy, particularly in Italy.

## 3 Background of the study

Green taxation and life expectancy have gained significant attention, particularly in the domains of environmental policy and public health. Governments use green taxation or eco-taxation as a policy tool to limit the activities that harm the environment by imposing taxes on such activities (25, 35, 36). These costs and regulations are enacted to discourage undesirable actions and reward those who are environmentally responsible (37). While the fundamental aim of green taxation is to protect the environment and promote sustainability, it can potentially impact public health and, particularly, life expectancy (38).

The baseline hypothesis of this study is that green taxation can have a significant positive influence on life expectancy. Such a narrative is also based on the premise that increasing the cost of pollution will encourage individuals and businesses to switch to less harmful alternatives (39, 40). This has the potential to improve public health and even extend people’s lifespans by curtailing pollution levels and encouraging environmentally responsible lifestyles (38).

However, there are several components and limitations to the aforementioned connection, all of which are influenced by a series of interdependent circumstances. The relationship between green taxation and life expectancy can be better understood with the help of economic dynamics (41). Sustainable practices can be encouraged through the use of taxation policies, and these policies also have favorable financial outcomes (42). These financial benefits may help reduce environmental damage and subsequently yield various health benefits, including longevity.

The relationship between green taxes and life expectancy also involves changes in people’s habits and ways of living (8). Individuals and businesses can be motivated to adjust their activities, particularly in the presence of a dynamic economic framework, such as through the use of green taxes (43). These behavioral changes could lead to healthier and more sustainable practices that promote greener lifestyles and enhance life expectancy.

Moreover, regional differences regarding green taxation policies are also important (44). Taxes are mostly diverse based on jurisdictions because of differences in policies and economic conditions. Therefore, it can be assumed that there are geographical differences in life expectancy, revealing potential variations in the effects of environmental taxes (45). Thus, we can hypothesize that:

H1: Environmental taxation may have a positive impact on life expectancy.

Research focusing on the complex nexus between green taxation and life expectancy has to incorporate the moderating role of public health expenditure to clearly understand the underlying mechanism of this association. Investing in public health could adjust the results of green taxation policies (46), which in turn affects life expectancy (47). Therefore, understanding this complex relationship is of considerable importance in today’s environmentally challenged societies.

It can be hypothesized that green taxation can increase life expectancy and that higher levels of public health expenditure can act as a moderator, enhancing these positive outcomes. Green taxation is established on the idea that it can improve public health by decreasing public exposure to dangerous pollutants and urging them to adopt more environmentally friendly practices (48, 49). However, the extent to which these advantages can be realized is determined by public health expenditures (50).

The term public health expenditure refers to the total amount of money spent on healthcare in the form of taxes and other government-level funds (51, 52). When it comes to addressing the health needs of a population, these resources are important, and they can be particularly useful in decreasing the adverse effects of environmental factors (53). It can also be expected that increases in public health expenditures will improve health care, lower its costs, and expand its accessibility (54, 55). Therefore, when public health facilities are easily accessible, the favorable economic impacts of green taxation on public health, such as lower healthcare expenditures and greater productivity, can get even more pronounced (56).

Public health budgets and the effects of green taxation on individual behavior are also closely linked (23, 57). Environmental taxation policies encourage eco-friendly behaviors, and a rise in public health expenditures can help finance the preventative measures, e.g., public awareness campaigns and health education (58, 59). By providing knowledge and resources for better living, public health programs may encourage individuals to adopt environmentally responsible behaviors (60, 61). This may result in healthier behavioral changes, which in turn can extend longevity.

Understanding the moderating role of public health expenditure may also require considering the regional differences regarding public health expenditure. The extent to which environmental taxation laws affect life expectancy can be influenced by how much funding is allocated to public health in certain regions or countries (62). Promoting public health and eventually contributing to longer and healthier lives, therefore, we can hypothesize that:

H2: Public health expenditures may have a positive moderating effect on the relationship between green taxation and life expectancy.

## 4 Methodology

### 4.1 Source

We use province-level data collected from the China Statistical Year Book published by the National Bureau of Statistics of China (NBS). The China Statistical Yearbook is a compilation of data that offers a complete picture of the country, provinces, and autonomous regions, Every year, the China Statistical Yearbook is published in September. Therefore, in this study, we only collect data until 2022 because the statistics for 2023 will be published in September 2024 (63). The data compiled by the NBS, which is renowned for its reliability, consistency, and accuracy, forms the basis of strategic decision-making and evidence-based policy-making. It offers an extensive range of economic, social, and demographic indicators. The final dataset consists of a diverse range of variables for 30 provinces and 19 years. Table 2 shows the list of provinces used to collect data from NBS.

**Table 2:** List of provinces.

Beijing	Jilin	Anhui	Hubei	Chongqing
Tianjin	Heilongjiang	Fujian	Hunan	Sichuan
Hebei	Shanghai	Jiangxi	Guangdong	Guizhou
Shanxi	Jiangsu	Shandong	Guangxi	Yunnan
Inner Mongolia	Zhejiang	Henan	Hainan	Xinjiang
Liaoning	Shaanxi	Gansu	Qinghai	Ningxia

### 4.2 Data

We use life expectancy as a dependent variable in this study. Life expectancy is a statistical measure that estimates the average number of years a person can expect to live. In China, such direct data is only calculated through census, which is conducted every 10 years. Therefore, we are unable to use this measure due to the unavailability of consistent data across the year. By considering such limitations, we use an indirect proxy to calculate life expectancy, i.e., the probability of dying. Previous studies, e.g., Roffia et al. (64) and Maiolo et al. (65), also used this proxy to measure life expectancy.

$$e_{0}=\frac{1}{\mu_{0}}$$

Whereas,

$$e_{0}=\mathrm{Life}\;\mathrm{Expectancy}$$

$$\mu_{0}=\mathrm{Central}\;\mathrm{Death}\;\mathrm{Rate}$$

The independent variable in this study is green taxation. Literature has provided various measuring tactics to calculate green taxation, e.g., environmental performance indicators, energy consumption, shifts in economic activities, etc. However, contrary to the said indicators, we follow the study of Fang et al. (66) and use environmental protection taxes as a proxy for green finance.

We employ public health expenditures as a moderating variable. Many different methods of measuring have been presented in the literature to calculate the said variable. These methods include government spending on hospitals, doctors, paramedics, etc. On the other hand, in contrast to the aforementioned indications, we adhere to the research conducted by Chipunza and Nhamo (56) and use total governmental health expenditures.

The following are the key control variables that are used to investigate the relationship between green taxation, public health expenditures, and life expectancy.

- Gross regional product: Province-level GRP
- Employment level: Total number of employed persons in the province
- Inflation: Consumer price index (CPI) in the province
- Individual income: *Per capita* income in the province
- Natural disasters: Total affected population in the province by natural disasters
- Population aging: Old age dependency ratio

### 4.3 Model

By considering the nature of the dataset and testing the aforementioned research hypotheses, we construct two empirical models. The first empirical model (1) quantifies the direct impact of green taxation on life expectancy. Similarly, our second empirical model (2) estimates the moderating relationship of public health expenditure on the nexus between green taxation and life expectancy.

(1) $$\begin{array}{l} \mathrm{Life}\;\mathrm{Expectanc}y_{kt}=\beta_{0}+\beta_{1}\mathrm{Green}\;\mathrm{Taxatio}n_{kt}\\ +\sum_{j=1}^{05}\beta_{j}\;\mathrm{Control}\;\mathrm{Variable}s_{kt}+e_{kt} \end{array}$$

(2) $$\begin{array}{l} \mathrm{Life}\;\mathrm{Expectanc}y_{kt}=\\ \beta_{0}+\beta_{1}\mathrm{Green}\;\mathrm{Taxatio}n_{kt}+\beta_{2}\mathrm{Public}\;\mathrm{Health}\;\exp_{kt}\\ +\beta_{3}\left(\mathrm{Green}\;\mathrm{Taxatio}n_{kt}\;X\;\mathrm{Public}\;\mathrm{Health}\;\exp_{kt}\right)+\sum_{j=1}^{05}\beta_{j}\\ \mathrm{Control}\;\mathrm{Variable}s_{kt}+e_{kt}\; \end{array}$$

The aforementioned Equations (1) and (2) represent regression models where the dependent variable is $\mathrm{Life}\;\mathrm{Expectanc}y_{kt}$ and the independent variable is $\mathrm{Green}\;\mathrm{Taxatio}n_{ik}$ . However, in Equation (2), particularly, we have a moderating variable that is constructed through an interactive term, i.e., $\mathrm{Green}\;\mathrm{Taxatio}n_{kt}\;X\;\mathrm{Public}\;\mathrm{Health}\;\exp_{kt}$ . Lastly, in both the empirical models, we have $\mathrm{Control}\;\mathrm{Variable}s_{kt}$ e.g., gross regional product, employment level, inflation, individual income, natural disasters, and population aging. In these contexts, *k* denotes individual provinces, and *t* denotes time. $\beta_{0}$ is an intercept term representing the expected value of life expectancy when all other variables become zero. e is an error term, representing the difference between the observed value of life expectancy and the value predicted by the models. It captures the effects of unobserved factors and random variation.

## 5 Empirical analysis and discussion

Table 3 presents the descriptive statistics (total number of observations, mean value, standard deviation, minimum and maximum values) of all the variables used in this study. This table summarizes the variables for better understanding. Particularly, the variable of life expectancy represents a mean value of 0.1494 with a standard deviation of 0.0262. Green taxation and public health expenditures have average values of 6.4134 and 6.3920, respectively, and their standard deviations are 7.2447 and 0.9895, respectively. The different control variables show the average value of gross regional productivity (10.0610), employment level (6.0083), inflation (4.6250), individual income (10.3283), natural disasters (5.2040), and population aging (3.2749). The standard deviations of the said indicators are gross regional productivity (0.9666), employment level (0.8637), inflation (0.0072), individual income (0.3282), natural disasters (1.6181), and population aging (2.4981).

**Table 3:** Descriptive statistics.

Variable	Observations	Mean	SD	Min	Max
Life expectancy	570	0.1494	0.0262	0.1094	0.2247
Green taxation	570	6.4134	7.2447	0.1430	40.9608
Public health expenditures	570	6.3920	0.9895	4.5246	14.9536
Gross regional productivity	570	10.0610	0.9666	7.3450	11.7685
Employment level	570	6.0083	0.8637	3.6082	7.6549
Inflation	570	4.6250	0.0072	4.6062	4.6415
Individual income	570	10.3283	0.3282	9.7577	11.2849
Natural disasters	570	5.2040	1.6181	−0.6931	7.8035
Population aging	570	3.2749	2.4981	0.0175	10.6400

The correlation coefficients of the variables are measured and explained in Table 4. The results demonstrate that the variables used for the analysis did not have any multicollinearity issues.

**Table 4:** Correlation.

	LE	GT	PHE	GRP	EMP	INF	II	ND	PA
LE	1.000								
GT	0.155	1.000							
PHE	0.064	0.304	1.000						
GRP	0.177	0.483	0.603	1.000					
EMP	0.113	0.444	0.597	0.677	1.000				
INF	−0.082	0.091	0.003	0.049	0.102	1.000			
II	0.164	0.201	0.457	0.504	0.462	−0.091	1.000		
ND	−0.397	0.105	0.049	0.346	0.356	0.136	−0.453	1.000	
PA	0.102	0.037	0.008	−0.073	−0.024	−0.021	−0.023	0.108	1.000

LE, life expectancy; GT, green taxation; PHE, public health expenditures; GRP, gross regional product; EMP, employment level; INF, inflation; II, individual income; ND, natural disaster; PA, population aging.

We incorporate the ordinary least square regression technique as a baseline tool To quantify the relationship between green taxation and life expectancy. However, as reported in Table 5, the results are statistically insignificant and misleading. One of the reasons for such deceptive results is the issue of unobserved heterogeneity.

**Table 5:** Base line regression (hypothesis—1).

Dependent variable = life expectancy	Model—1	
	Ordinary least square (OLS)	
	Province-year panel dataset	
	Coefficients	t-stats
Green taxation	−0.0012	(−0.75)
Gross regional productivity	−0.0356	(−9.00)***
Employment level	0.0367	(9.93)***
Inflation	0.0013	(2.57)***
Individual income	0.0221	(5.20)***
Natural disasters	−0.0021	(−2.50)***
Population aging	0.0005	(1.48)
Constant	−0.0483	(−0.74)
Number of observations	570	
F-Stats	30.50	
Prob. > F	0.000	
R-squared	0.2753	
Adj. R-squared	0.2662	

***p < 0.01.

Similar to the aforementioned prevailing statistical concern, i.e., the heterogeneity issue, the results documented in Table 6 to estimate the moderating influence of public health expenditure on the nexus between life expectancy and green taxation are ambiguous and need future investigation.

**Table 6:** Base line regression (hypothesis—2).

Dependent variable = life expectancy Moderating variable = public health expenditures	Model—2	
	Ordinary least square (OLS)	
	Province-year panel dataset	
	Coefficients	t-stats
Green taxation	0.0024	(1.57)
Public heath expenditure	0.0034	(1.02)
Green taxation × public heath expenditure	−0.0002	(−1.67)*
Gross regional productivity	0.0384	(7.78)***
Employment level	0.0369	(9.51)***
Inflation	0.0013	(2.59)***
Individual income	0.0211	(4.62)***
Natural disasters	−0.0024	(2.73)***
Population aging	0.0005	(1.54)
Constant	−0.0490	(−0.74)
Number of observations	570	
F-Stats	24.16	
Prob. > F	0.0000	
R-squared	0.2797	
Adj. R-squared	0.2681	

***p < 0.01, *p < 0.10.

To address the presence of heterogeneity in both empirical models, we use the fixed effect regression technique. Panel data, which collects observations over multiple periods and for multiple entities, makes fixed effect models particularly useful. Moreover, for selection between fixed effect and random effect regression, we use the Hausman test. According to the results stated in Table 7, the *p*-value of chi-square is significant; therefore, we can reject the null hypothesis and choose an alternate hypothesis, i.e., a fixed effects model is preferred. Moreover, the regression results show a positive and statistically significant impact of green taxation on life expectancy. These results verify our first hypothesis and are in line with the previous study of Tenytska and Palienko (67). These results suggest that environmental taxation escalates the cost of environmental damage, which eventually encourages individuals and businesses to switch to less harmful alternatives. Such a protective approach will help advance longevity.

**Table 7:** Advanced regression (hypothesis—1).

Dependent variable = life expectancy	Model—1	
	Fixed effect	
	Province-year panel dataset	
	Coefficients	t-stats
Green taxation	0.0026	(2.47)***
Gross regional productivity	0.0227	(4.52)***
Employment level	0.0356	(8.41)***
Inflation	0.0005	(1.61)*
Individual income	0.0019	(2.72)***
Natural disasters	−0.0421	(7.69)***
Population aging	0.0031	(2.04)***
Constant	0.0815	(1.98)**
Number of observations	570	
Number of groups	30	
F-Stats	46.91	
Prob. > F	0.000	
R-sq. (within)	0.3812	
R-sq. (between)	0.0773	
R-sq. (overall)	0.0240	
Hausman test		
Chi^2^	54.05	
Prob. > Chi^2^	0.0000	

***p < 0.01, **p < 0.05, *p < 0.10.

Likewise, to test our second hypothesis (i.e., public health spending moderates the link between green taxes and life expectancy), we again run the Hausman test, and based on the significance *p*-value, we choose the fixed effect moderation model. Furthermore, the technique also helps us deal with the problem of heterogeneity. The results listed in Table 8 show that green taxation, public health expenditures, and the interacting terms of both variables all have a positive and statistically significant impact on life expectancy. These outcomes support our narrative and confirm the partial moderating impact of public health expenditures. Moreover, these results provide a significant understanding that environmental taxation increases life expectancy and that higher levels of public health expenditure can operate as moderators, enhancing these positive outcomes. Increases in public health expenditures will eventually improve health care outcomes, lower its associated costs, and improve the availability of public health facilities. Thus, the accessibility of public healthcare reinforces the economic benefits of green taxation for an improved life expectancy.

**Table 8:** Advanced regression (hypothesis—2).

Dependent variable = life expectancy Moderating variable = public health expenditures	Model—2	
	Fixed effect—moderation	
	Province-year panel dataset	
	Coefficients	t-stats
Green taxation	0.0017	(2.48)***
Public health expenditures	0.0284	(7.84)***
Green taxation × public health expenditures	0.0002	(1.63)*
Gross regional productivity	0.0042	(0.68)
Employment level	0.0319	(7.73)***
Inflation	−0.0522	(−9.29)***
Individual income	0.0005	(1.79)*
Natural disasters	−0.0007	(−1.11)
Population aging	0.0013	(2.62)***
Constant	0.2378	(5.39)***
Number of observations	570	
Number of groups	30	
F-Stats	48.05	
Prob. > F	0.0000	
R-sq. (within)	0.4489	
R-sq. (between)	0.1231	
R-sq. (overall)	0.0303	
Hausman test		
Chi^2^	158.06	
Prob. > Chi^2^	0.0000	

***p < 0.01, *p < 0.10.

Although fixed-effect regression models are efficient at controlling for heterogeneity, endogeneity is a valid concern, particularly in the context of a panel dataset. Therefore, we used the system GMM method to control for the panel-specific endogeneity problems that may be present in the aforementioned models. Moreover, for the validation of GMM models, we have also used the Sargan test for over-identifying restrictions. The Sargan test’s *p*-value suggests that our over-identified restrictions in the models are valid. The empirical results reported in Table 9 suggest a positive impact of green taxation on life expectancy. Similarly, the findings also verify the positive partial moderation of public health on the nexus between green taxation and life expectancy (Table 10). Hence, our baseline results remains unchanged.

**Table 9:** Advanced regression (hypothesis—1).

Dependent variable = life expectancy	Model—1	
	System GMM	
	Province-year panel dataset	
	Coefficients	t-stats
Green taxation	0.0071	(2.52)***
Gross regional productivity	0.0395	(8.52)***
Employment level	0.0344	(5.62)***
Inflation	−0.0005	(−1.66)*
Individual income	0.0257	(4.43)***
Natural disasters	−0.0012	(−0.95)
Population aging	0.0007	(3.95)***
Constant	0.0109	(0.25)
Number of observations	570	
Number of groups	30	
Wald chi^2^	162.04	
Prob. > chi^2^	0.000	
Sargan test (*p*-value)	0.204	

***p < 0.01, *p < 0.10.

**Table 10:** Advanced regression (hypothesis—2).

Dependent variable = life expectancy Moderating variable = public health expenditures	Model—2	
	System GMM—moderation	
	Province-year panel dataset	
	Coefficients	t-stats
Green taxation	0.0078	(1.70)*
Public health expenditures	0.0138	(2.29)***
Green taxation × public health expenditures	0.0009	(1.76)*
Gross regional productivity	0.0429	(3.56)***
Employment level	0.0329	(3.36)***
Inflation	−0.0012	(−3.69)***
Individual income	0.0149	(1.42)
Natural disasters	−0.0039	(−2.02)**
Population aging	0.0007	(1.77)*
Constant	0.0164	(0.19)
Number of observations	570	
Number of groups	30	
Wald chi^2^	104.84	
Prob. > chi^2^	0.000	
Sargan test (*p*-value)	0.999	

***p < 0.01, **p < 0.05, *p < 0.10.

Green or environmental taxation is an effective approach that provides benefits for sustainable practices, reduces environmentally harmful activities, and thus enhances life expectancy. One apparent benefit of green taxation is its ability to reduce pollution. High taxes on harmful practices, such as the ignition of fossil fuels, have the potential to decrease emissions and foster air quality. Improved air quality is associated with fewer cases of respiratory disease and cardiovascular issues, both of which add years to a person’s life expectancy (49).

Moreover, green taxation has the potential to encourage investment, particularly in the renewable energy and health sectors. By increasing the cost of fossil fuels, competent authorities provide benefits to businesses and individuals who switch to cleaner energy alternatives. Additionally, to reduce emissions, the implementation of sustainable alternatives such as solar power and electric vehicles (EVs) contributes to better health consequences. These sustainable alternatives frequently generate employment opportunities and foster economic growth, thus facilitating enhanced healthcare accessibility and improved quality of life, both of which contribute to an extended lifespan.

Green taxation could also help to enhance life expectancy by addressing environmental challenges, such as climate change. Such a reduction in carbon emissions and encouraging healthier practices could subsequently contribute to reducing global warming, improving food security, diminishing climate-related health threats, and decreasing extreme weather conditions. Mostly, the application of green taxation aids a variety of objectives: it endorses sustainable technology, reduces pollution, and mitigates environmental hazards; therefore, it contributes to the long-term progress of well-being and prosperity.

The moderating role of public health expenditure, particularly in the context of the positive relationship between green taxation and life expectancy, is significant. The implementation of green taxation policies has the potential to encourage sustainable practices and reduce pollution, thereby positively affecting public health and extending life expectancy. Governmental investment in the public health sector could significantly improve the efficiency of the said measures. We can further boost the benefits of environmental taxation, disease control, and health elevation by allocating additional reserves toward public health. These steps could lead to greener environments and healthier lifestyles.

In countries with resilient healthcare systems, higher health expenditures can ensure that the population receives needed healthcare services. This can aid in early diagnosis and improve health issues, thereby extending life expectancy. Furthermore, allocating funds to public health can address health disparities and target deprived communities, thus tackling the social aspects that influence health outcomes. Ensuring fair distribution of the benefits of green taxation is crucial. To summarize, green taxation has the potential to improve life expectancy. However, the level of public health expenditure influences the extent to which this occurs. This guarantees the optimization and equitable distribution of the benefits of green taxation to those most in need, leading to significant and equitable increases in life expectancy (62).

## 6 Conclusion

This study aims to explore the impact of green taxation on life expectancy in China. Moreover, we also examine the moderating role of public health expenditures in influencing the proposed association. As the second largest world economy, Chinese economy is one of the most vibrant and emerging economies globally. To explore the said narrative, yearly provincial data was obtained from the National Bureau of Statistics—China. By employing robust econometric approaches such as fixed effect and system GMM regression estimates along with diverse control variables, the finding of the study provides significant supportive evidence toward the research hypotheses and testifies that green taxation enhances life expectancy in the context of China. Moreover, public health expenditures exhibits a favorable influence and partially moderate the said relationship. These results are robust even by choosing different proxies of public health expenditures.

This study significantly contributes to the ongoing discussion in the fields of environmental and public health economics by offering valuable insights for both academic researchers and policymakers. By specifically examining the impact of green taxation on life expectancy and considering the moderating role of public health expenditures, particularly within the context of China’s dynamic and rapidly growing economy, this research addresses a critical gap in the literature. Academic researchers can benefit from the findings by deepening their understanding of the nexus between green taxation, public health expenditures, and life expectancy and fostering further exploration in the said fields.

The policy implications of this study propose the importance of implementing green taxation to enhance life expectancy and the role of public health expenditure in strengthening the relationship between green taxation and life expectancy. For policymakers, this study offers fresh insights and directions for the development of sustainable environmental and public health policies. The positive association between green taxation and life expectancy, linked with the moderating effect of public health expenditures. Hence this research provides a base for formulating strategies that balance economic growth with environmental sustainability and advancement in public health.

Although this study has significantly contributed to exploring the relationship between green taxation, public health expenditures, and life expectancy in China, there are certain limitations as well. First, we use provincial data across years, which cannot observe the variations within cities and regions. Moreover, this paper focuses on China, which limits its generalizability to other countries with different cultures and institutions. Future studies could address these limitations by including longitudinal data (cross-countries) and extending the analysis to global settings. Overall, addressing these limitations and exploring the proposed research directions may help to develop a more detailed understanding of the interplay between environmental policies, healthcare expenditures, and life expectancy.

## Data availability statement

Publicly available datasets were analyzed in this study. This data can be found at: https://data.stats.gov.cn/english.

## Author contributions

DZ: Conceptualization, Data curation, Formal analysis, Funding acquisition, Investigation, Methodology, Project administration, Resources, Software, Supervision, Validation, Visualization, Writing – original draft, Writing – review & editing.
